# Apoptotic Markers Are Increased in Epilepsy Patients: A Relation with Manganese Superoxide Dismutase Ala16Val Polymorphism and Seizure Type through IL-1*β* and IL-6 Pathways

**DOI:** 10.1155/2020/6250429

**Published:** 2020-03-06

**Authors:** Aline Kegler, Ana Letícia Fornari Caprara, Eduardo Tanuri Pascotini, Josi Arend, Patricia Gabbi, Marta M. M. F. Duarte, Ana Flavia Furian, Mauro Schneider Oliveira, Luiz Fernando Freire Royes, Michele Rechia Fighera

**Affiliations:** ^1^Centro de Ciências da Saúde, Departamento de Neuropsiquiatria, Universidade Federal de Santa Maria, RS, Brazil; ^2^Centro de Ciências Naturais e Exatas, Programa de Pós-Graduação em Ciências Biológicas: Bioquímica Toxicológica, Universidade Federal de Santa Maria, RS, Brazil; ^3^Centro de Ciências da Saúde, Programa de Pós-Graduação em Farmacologia, Universidade Federal de Santa Maria, RS, Brazil; ^4^Centro de Educação Física e Desportos, Laboratório de Bioquímica do Exercício (BIOEX), Universidade Federal de Santa Maria, RS, Brazil

## Abstract

The MnSOD Ala16Val single nucleotide polymorphism (SNP) has been associated with different diseases. However, there are scarcely studies relating this SNP in epilepsy, a neurologic disease that involves some interacting pathways, such as apoptotic and inflammatory factors. In this sense, we decided to investigate the relationship of MnSOD Ala16Val SNP with apoptotic markers in epilepsy and its relation with inflammatory pathway and seizure type. Ninety subjects were evaluated (47 epilepsies; 43 controls) by questionnaires and laboratorial exams. We observed a higher percentage of VV genotype in the epilepsy group when compared to the control group. IL-1*β*, IL-6, caspase-1, and caspase-3 levels were increased in the epilepsy group (VV genotype). Furthermore, an important correlation between IL-1*β* vs. caspase-1 and IL-6 vs. caspase-3 was observed in the epilepsy group (VV genotype). The epilepsy group which presented generalized seizures also demonstrated a positive correlation between IL-1*β* vs. CASP1 and IL-6 vs. CASP3. Thus, it is a plausible propose that epilepsy patients with VV genotype and generalized seizures present a worse inflammatory and apoptotic status. Our findings suggest that the knowledge of MnSOD Ala16Val polymorphism existence is important to evaluate molecular mechanisms associated to seizure and improve the treatment of these patients.

## 1. Introduction

Epilepsy is one of the most common neurological disorders [1] characterized by an enduring predisposition to generate seizures [2] affecting more than 65 million people worldwide [3]. Despite progress in pharmacological and surgical treatments of epilepsy, it is not clear about the processes leading to the generation of seizures and about the mechanisms; whereby, a healthy brain is rendered epileptic [4]. Apoptosis [5], neuroinflammation [4], and oxidative stress [6] are some relevant factors implicated in epilepsy pathophysiology. Many works have acknowledged the role of neuroinflammation in the pathogenesis of seizures, but little is known about the mechanisms that start the inflammatory process in epilepsy [7]. The microglia constitute the primary CNS immune cells and are quickly activated in response to an insult. However, the excessive activation of microglia may be harmful, promoting the development of neuronal diseases by producing large amounts of inflammatory molecules, such as IL-6 [8], IL-1*β*, and reactive oxygen species (ROS) [9]. In epilepsy, there is a complex cascade of molecular and cell mechanisms involved in excitotoxicity [10], oxidative stress [11], and inflammation [4] beyond cytotoxicity mediated by cytokines [8] and cell death pathway activation [12]. In fact, when the brain is affected by brain diseases (i.e., epilepsy), the microglia cells are activated [13], and this activation may lead to production of inflammatory cytokines as IL-*β* [14] and IL-6 [15]. Interestingly, some antioxidant molecules were reported to decrease the levels of proinflammatory mediators by scavenging ROS [16]. Therefore, the redox balance is thought to regulate a series of neuroinflammatory processes mediated by microglia [9]. Manganese superoxide dismutase (MnSOD) antioxidant enzyme is the only known major defense against reactive oxygen species within mitochondria [17]. Furthermore, MnSOD is reportedly induced in the CNS under inflammatory conditions [9]. Regarding the relevance of MnSOD, numerous factors can impact on the effectiveness of antioxidant enzymes, including enzymatic polymorphism [18]. Two main MnSOD SNPs have been described in the literature, one of which is Ala16Val [17]. The change of alanine (Ala) to valine (Val) at the 16^th^ amino acid (Ala16Val) of the signal sequence of MnSOD is suggested to change the structure of the protein. The alanine-to-valine substitution produces a *β*-sheet secondary structure instead of an *α*-helix structure, decreasing the enzyme transport efficiency into the mitochondria and compromising the antioxidant potential [19]. The Ala16Val MnSOD SNPs generate three possible genotypes: AA, AV, and VV. Sutton et al. [20] reported that the Val allele results in reduced expression and production of an unstable mRNA, affecting the import of SOD2 into the mitochondria. Accordingly, Montano et al. [21] demonstrated that the VV and AV peripheral blood mononuclear cells (PBMC) presented increased levels of inflammatory cytokines as IL-1*β* and IL-6.

There are studies reporting the association of inflammatory and apoptotic parameters with MnSOD Ala16Val SNP in diseases as stroke [22, 23] and cancer [24]. However, there are few evidences of involvement of Ala16Val MnSOD SNP in epilepsy [25]. In this sense, the aim of this study was to investigate caspases' activation and relationship between Ala16Val MnSOD SNP with interleukins in epilepsy patients. Additionally, we have also investigated the relation of seizure type (partial or generalized) and duration time (minutes) of seizures with parameters aforementioned. This study is of such interest in view that an enzymatic polymorphism has an important role in inflammatory and apoptotic pathway, and the obtained results can contribute to a better understanding about the epilepsy disease and its mechanisms, maybe providing support to novel approaches of pharmacotherapy.

## 2. Materials and Methods

### 2.1. Study Design


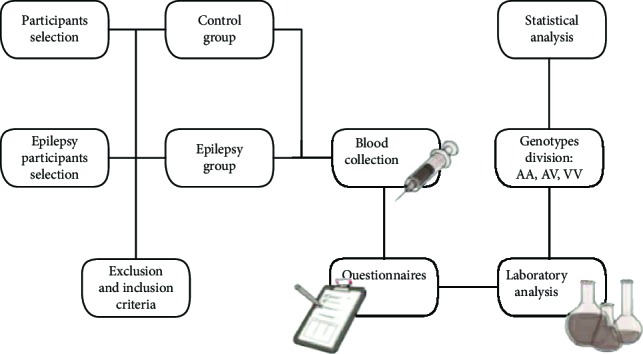


### 2.2. Participants

We performed a case-control study and a total of 90 subjects were recruited and allocated into two groups: epilepsy group (*n* = 47) and control group (healthy subjects, *n* = 43). No etiology was found after detailed history, physical, laboratory, and imaging studies. Major exclusion criteria were history of autoimmune, liver, kidney, and inflammatory diseases; allergic response; immune deficiency disorder; diabetes, psychiatric illness; malignancy; smoking; or a systemic or central nervous system (CNS) infection 2 weeks before sample collection. The study protocol was approved by the local institutional review boards at the authors' affiliated institutions. Informed written consent was obtained from all the subjects or their legal surrogates. The work described has been carried out in accordance with The Code of Ethics of the World Medical Association (Declaration of Helsinki).

### 2.3. Epilepsy Group

The epilepsy patients were recruited from the University Hospital of Santa Maria and invited to volunteer for the study. Epilepsy was diagnosed by two experienced neurologists according to the 2010 International League Against Epilepsy (ILAE) Classification, revised in 2017 ([26]; Fisher et al., 2017; Scheffer et al., 2017). All patients were evaluated for seizure frequency using seizure diaries [27]. The seizure type from epilepsy group (*n* = 47) was confirmed through interviews with the patients and their relatives as well as EEG analysis and tomography or magnetic resonance imaging (MRI). For a diagnosis of generalized epilepsy, the patient would typically show generalized spike-wave activity on EEG. Individuals with generalized epilepsies may have a range of seizure types including absence, myoclonic, atonic, tonic, and tonic–clonic seizures. The diagnosis of generalized epilepsy is made on clinical grounds, supported by the finding of typical interictal EEG discharges. Caution needs to be exercised for a patient with generalized tonic–clonic seizures and a normal EEG. In this case, supportive evidence would need to be presented to make a diagnosis of generalized epilepsy, such as myoclonic jerks or a relevant family history. Focal epilepsies include unifocal and multifocal disorders as well as seizures involving one hemisphere. A range of seizure types can be seen including focal aware seizures, focal impaired awareness seizures, focal motor seizures, focal nonmotor seizures, and focal to bilateral tonic–clonic seizures. The interictal EEG typically shows focal epileptiform discharges, but the diagnosis is made on clinical grounds, supported by EEG findings. The term “unknown” is used to denote where it is understood that the patient has epilepsy, but the clinician is unable to determine if the epilepsy type is focal or generalized because there is insufficient information available.

In our study, forty-five patients were in remission except for two patients who were diagnosed with refractory epilepsy. All epilepsy patients had normal neurological examinations except for one who presented tetra paresis secondary to spinal cord lesion. All epilepsy had normal 1.5 T MRI; one patient had right and left hippocampal sclerosis.

### 2.4. Study Variables

Sex (dichotomous): male and female

Age (quantitative): years

Antiepileptic drugs (quantitative): number of drugs used for each patient

MnSOD Ala16Val genotype AA, AV, and VV (quantitative): frequencies (%)

Epilepsy type (dichotomous): generalized, focal, and unknown

Protein carbonyl (quantitative): nmol/mg protein

SOD2 activity (quantitative): U/mg hemoglobin

IL-1*β* (quantitative): pg/mL

IL-6 (quantitative): (pg/mL)

Caspase-3 (quantitative): mg/mL

Caspase-1 (quantitative): mg/mL

### 2.5. Laboratory Analyses

Samples were collected at least 7 days from the last seizure attack (Mao et al., 2013). After 12 h of overnight fasting, blood samples were collected by venipuncture using purple, green, and red top Vacutainer® (BD Diagnostics, Plymouth, UK) tubes with ethylenediamine tetra acetic acid (EDTA), heparin, or no anticoagulants, respectively. The specimens were routinely centrifuged within 1 h of collection for 15 min at 2500 g and aliquots of the serum samples, and the supernatant was saved and stored at -80°C for subsequent laboratory analysis, according to specific methods.

### 2.6. Protein Carbonyl (PC)

The analysis of protein carbonyl was in accordance with [28].

### 2.7. Manganese Superoxide Dismutase (MnSOD)

The manganese superoxide dismutase activity was performed in accordance with [29].

### 2.8. Caspase Determination

Caspase-1 and caspase-3 activities were determined by Fluorimetric Assay Kits (BioVision, Mountain View, CA). The fluorescence intensity was recorded at wavelength of 400 nm for excitation and at wavelength of 505 nm for emission for both. The activity was then calculated as fluorescence intensity (FI)/min/mL = ΔFlt/(t × v), where *Δ*Flt is the difference in fluorescence intensity between time zero and time *t* minutes, *t*is the reaction time in min, and *v*is the volume of sample in mL.

### 2.9. Cytokine Determination

The cytokines were assessed by ELISA using commercial kits for human IL-1*β* and IL-6 (eBioscience, San Diego, USA).

### 2.10. DNA Damage

The alkaline DNA comet assay as described by Pereira. Genomic DNA was isolated from peripheral blood leukocytes using a DNA Mini Kit Purification (Mo Bio).

### 2.11. MnSOD Ala16Val Genotyping

Genomic DNA was isolated from peripheral blood leukocytes using a DNA Mini Kit Purification (Mo Bio). MnSOD Ala16Val SNP was detected by PCR-RFLP according to Taufer et al. PCR amplifications were performed in a total volume of 50 *μ*l containing 5 *μ*l of 10x buffer, 1 *μ*l of 25 mM MgCl2, 1.25 *μ*l of 10 mM dNTP, 0.5 *μ*l of Taq polymerase (Gibco Inc., Co.), 1 *μ*l of each primer (40 pmol), 3 *μ*l of genomic DNA (0.25 *μ*g), and 34.5 *μ*l of ddH2O. The amplification primers (Gibco Inc., Co.) for a 110 bp fragment of the human MnSOD gene were 5′-ACCAGCAGGCAGCTGGCGCCGG-3′ (sense strand) and 5′-GCGTTGATGTGAGGTTCCAG-3′ (antisense strand) with the following thermocycler parameters: an initial cycle of 95°C for 5 min followed by 35 cycles at 95°C for 1 min and 61°C for 1 min. The final cycle was followed by an extension period of 2 min at 72°C. The PCR product (10 *μ*l) was digested with Hae III (15 U; 37°C; 6 h; Gibco Inc., Co.). Digested products (23 and 85 bp) were visualized on a 4% agarose gel (Amersham Biosciences Inc., Co.) stained with ethidium bromide. A mutation was introduced by a primer mismatch to create a restriction cut site for Hae III in the -9 codon, and the following genotypes were observed: -9Ala/Ala (23 and 85 bp); -9Ala/Val (23, 85, and 110 bp); and -9Val/Val (110 bp).

### 2.12. Sample Size

As there is no comparison in the literature of apoptotic, inflammatory, and oxidative levels with MnSOD polymorphism in epilepsy patients, an adequate calculation if the sample size is not possible. Considering a significant difference of a standard deviation between the two groups and using the PEPI software, considering a study power of 90% and an alpha error of 0.05, 46 patients would be needed.

### 2.13. Statistical Methods

Data were analyzed by analysis of variance (two-way ANOVA) followed by Tukey's multiple comparison test. Statistical analysis was performed using the SPSS (Statistical Package for the Social Sciences) software in a PC-compatible computer. Correlation analyses were carried out using the Pearson correlation coefficient. Statistical significance was assumed when *p* < 0.05. Chi-square test was used to calculate sex, age, and genotype frequencies.

## 3. Results

Baseline characteristics of the participants are described in Table 1. According to Chi-square analysis, no statistically difference was observed between the epilepsy group and control group relating with sex (*p* = 0.5) and age (*p* = 0.6).

Analysis of the Ala16Val MnSOD gene yielded three variants of the genotype: AA (wild type), AV (heterozygous), and VV (homozygous). The Ala16Val MnSOD genotype frequencies were calculated and are presented in Table 2. In the epilepsy group, the genotype frequencies were 31.9% for AA, 21.2% for AV, and 46.8% for VV. The frequencies for AA, AV, and VV genotypes were 39.5%, 32.5%, and 27.9%, respectively, in the control group. According to Chi-square analysis, no statistically difference in Ala16Val MnSOD genotype frequencies was observed (*p* = 0.1).

### 3.1. Protein Carbonyl (PC)

**Figure 1:**
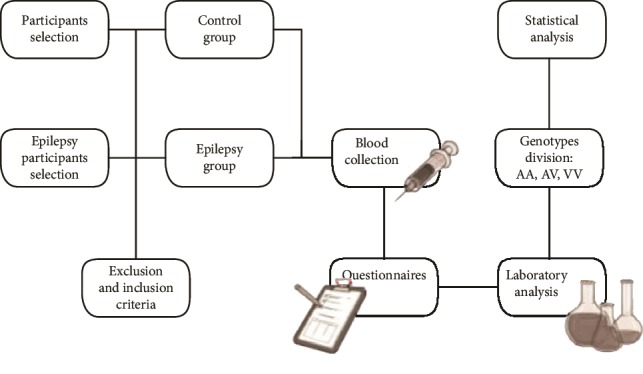
Comparison of Ala16Val MnSOD polymorphism genotypes (AA, AV, and VV) from control and epilepsy groups in relation to oxidative stress biomarker. The epilepsy group presented increased levels of protein carbonyl when compared to control group. ^∗^*p* < 0.05 when compared to respective control group.

A two-way ANOVA demonstrated increased protein carbonyl levels in the epilepsy group when compared to the control group (*F*(1, 84) = 36.48, *p* < 0.0001). *Post hoc* analysis with Tukey's test for multiple comparisons revealed increased levels of PC in the epilepsy group (AA, AV, and VV genotypes) when compared to their genotypes from the control group, respectively (Figure 1).

### 3.2. Manganese Superoxide Dismutase (MnSOD)

**Figure 2:**
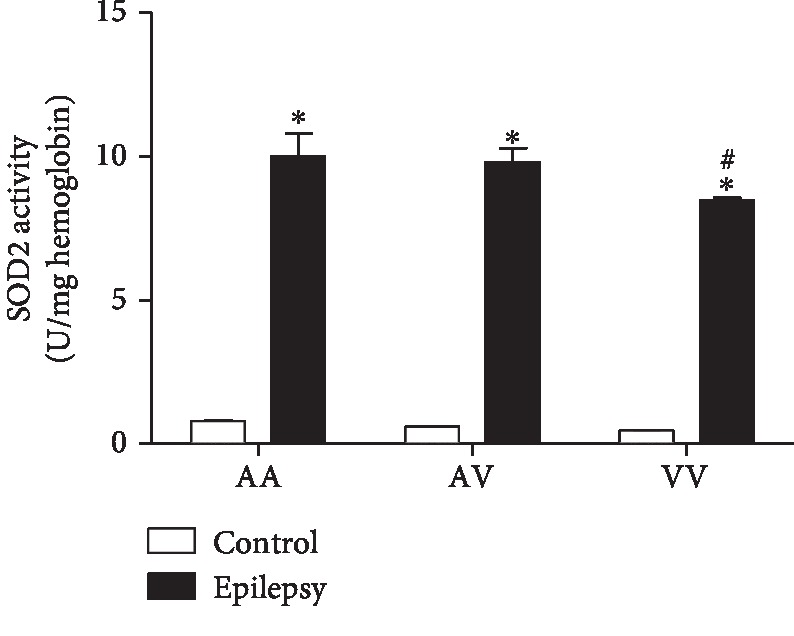
Comparison of Ala16Val MnSOD polymorphism genotypes (AA, AV, and VV) from control and epilepsy groups in relation to SOD2 activity. The epilepsy group presented increased SOD2 activity when compared to the respective control group. The epilepsy group (VV) presented a decreased SOD2 activity when compared to epilepsy group (AA). ^∗^*p* < 0.05 when compared to respective control group; ^#^*p* < 0.05 when compared to the epilepsy group (VV vs. AA).

A two-way ANOVA demonstrated increased MnSOD enzyme activity in the epilepsy group when compared to the control group (*F*(1, 81) = 617.5, *p* < 0.0001). *Post hoc* analysis with Tukey's test for multiple comparisons revealed increased MnSOD activity in the epilepsy group (AA, AV, and VV genotypes) when compared to their genotypes from the control group, respectively. Furthermore, in the epilepsy group, the homozygous VV genotype presented decreased enzyme activity when compared to AA genotype (Figure 2).

### 3.3. IL-1*β*

**Figure 3:**
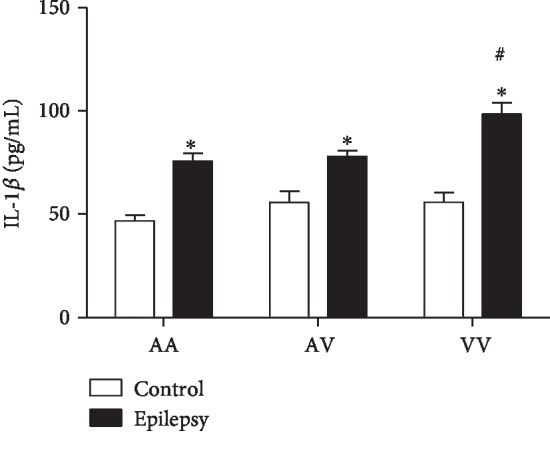
Comparison of Ala16Val MnSOD polymorphism genotypes (AA, AV, and VV) from control and epilepsy groups in relation to IL-1*β*. The epilepsy group presented increased levels of IL-1*β* when compared to control group. ^∗^*p* < 0.05 when compared to respective control group; ^#^*p* < 0.05 when compared to the epilepsy group (VV vs. AV and AA).

A two-way ANOVA demonstrated increased IL-1*β* levels in the epilepsy group when compared to the control group (*F*(2, 85) = 6.2, *p* < 0.01). *Post hoc* analysis with Tukey's test for multiple comparisons revealed increased IL-1*β* levels in the epilepsy group (AA, AV, and VV genotypes) when compared to their genotypes from the control group, respectively. Furthermore, in the epilepsy group, the homozygous VV genotype presented increased levels when compared to AV and AA genotypes (Figure 3).

### 3.4. IL-6

**Figure 4:**
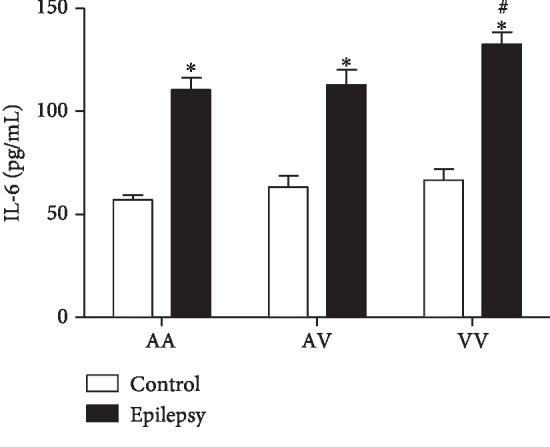
Comparison of Ala16Val MnSOD polymorphism genotypes (AA, AV, and VV) from control and epilepsy groups in relation to IL-6. The epilepsy group presented increased levels of IL-6 when compared to control group. ^∗^*p* < 0.05 when compared to respective control group; ^#^*p* < 0.05 when compared to the epilepsy group (VV vs. AA).

A two-way ANOVA demonstrated increased IL-6 levels in the epilepsy group when compared to the control group (*F*(2, 77) = 4.6, *p* < 0.05). *Post hoc* analysis with Tukey's test for multiple comparisons revealed increased IL-6 levels in the epilepsy group (AA, AV, and VV genotypes) when compared to their genotypes from the control group, respectively. Furthermore, in the epilepsy group, the homozygous VV genotype presented increased levels when compared to AA genotype (Figure 4).

### 3.5. Caspase-3

**Figure 5:**
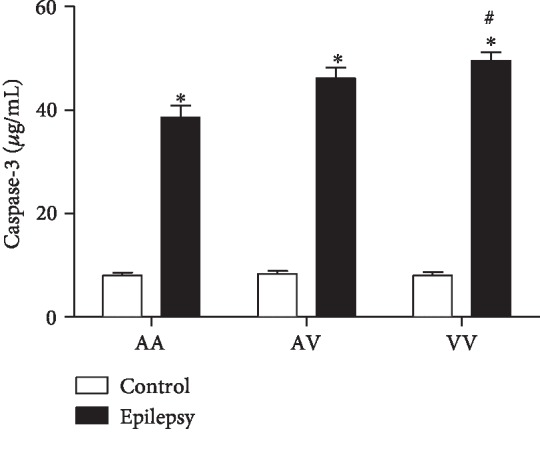
Comparison of Ala16Val MnSOD polymorphism genotypes (AA, AV, and VV) from control and epilepsy groups in relation to caspase-3. The epilepsy group presented increased levels of caspase-3 when compared to control group. ^∗^*p* < 0.05 when compared to respective control group; ^#^*p* < 0.05 when compared to the epilepsy group (VV vs. AA).

A two-way ANOVA demonstrated increased caspase-3 levels in the epilepsy group when compared to the control group (*F*(2, 77) = 6.9, *p* < 0.01). *Post hoc* analysis with Tukey's test for multiple comparisons revealed increased caspase-3 levels in the epilepsy group (AA, AV, and VV genotypes) when compared to their genotypes from the control group, respectively. Furthermore, in the epilepsy group, the homozygous VV genotype presented increased levels when compared to AA genotype (Figure 5).

### 3.6. Caspase-1

**Figure 6:**
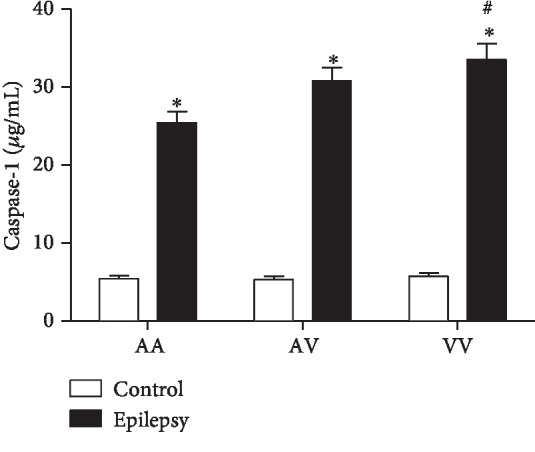
Comparison of Ala16Val MnSOD polymorphism genotypes (AA, AV, and VV) from control and epilepsy groups in relation to caspase-1. The epilepsy group presented increased levels of caspase-1 when compared to control group. ^∗^*p* < 0.05 when compared to respective control group; ^#^*p* < 0.05 when compared to the epilepsy group (VV vs. AA).

A two-way ANOVA demonstrated increased caspase-1 levels in the epilepsy group when compared to the control group (*F*(2, 77) = 3.8, *p* < 0.05). *Post hoc* analysis with Tukey's test for multiple comparisons revealed increased caspase-1 levels in the epilepsy group (AA, AV, and VV genotypes) when compared to their genotypes from the control group, respectively. Furthermore, in the epilepsy group, the homozygous VV genotype presented increased levels when compared to AA genotype (Figure 6).

### 3.7. Comet Assay

A two-way ANOVA demonstrated increased amount of DNA damage in the epilepsy group when compared to the control group (*F*(1, 84) = 1282, *p* < 0.0001). *Post hoc* analysis with Tukey's test for multiple comparisons revealed increased amount of DNA damage in epilepsy group (AA, AV, and VV genotypes) when compared to their genotypes from the control group, respectively (data not shown).

### 3.8. Correlations

#### 3.8.1. IL-1*β* vs. Caspase-1

Pearson's analysis demonstrated an interesting correlation between IL-1*β* and caspase-1 (*r* = 0.7, *p* < 0.001) in the epilepsy group (VV genotype) (Table 3).

#### 3.8.2. IL-6 vs. Caspase-3

Pearson's analysis demonstrated an interesting correlation between IL-6 and caspase-3 (*r* = 0.5, *p* < 0.05) in the epilepsy group (VV genotype) (Table 3).

#### 3.8.3. Seizure Type vs. Polymorphism

Pearson's analysis demonstrated in the epilepsy group which presented generalized seizures (VV genotype), an interesting correlation between inflammatory and apoptotic parameters: IL-1*β* vs. caspase-1 (*r* = 0.7, *p* < 0.05) and IL-6 vs. caspase-3 (*r* = 0.6, *p* < 0.05). Furthermore, the results demonstrated an increased in caspase-1 levels in the epilepsy group which presented generalized seizures (VV genotype) (*t* = 2.89, *p* < 0.05). The other parameters did not demonstrate significant alteration in relation to generalized or partial seizures (data not shown) (Table 3).

#### 3.8.4. Seizures' Duration Time (Minutes)

The statistical analysis revealed that the epilepsy group which presented generalized seizures (VV genotype) presented longer seizure time (minutes) than the epilepsy group which presented partial seizures (VV genotype) (*t* = 2.46, *p* < 0.05).

## 4. Discussion

The novel finding of the study is the influence of Ala16Val MnSOD gene polymorphism-VV genotype on inflammatory (IL-6, IL-1*β*), apoptotic (caspases -1 and -3) and antioxidant enzyme (MnSOD) in epilepsy. Of such interest, we observed an interesting correlation (caspase-1 vs. IL-1*β*) and (caspase-3 vs. IL-6) in VV epilepsy patients. Furthermore, the generalized seizures were impacted by the VV genotype in relation to the referred parameters and with relation to seizures' duration time. The burst firing neurons associated with epileptic discharges could lead to changes with events of cascades at the cellular level [30]. The complex mechanism of epileptogenesis remains largely unclear. However, oxidative stress by free radical generation does indeed play a role in mitochondrial dysfunction [31]. Furthermore, the oxidative stress can alter/influence factors leading to neuronal death, and, consequently the DNA damage [32]. The intense seizure activity can lead to cytotoxic effects mediated by oxidative stress. The superoxide anion (O_2_^−^) is the central mediator of oxidative stress, influencing both physiological and pathological processes [33]. While there are some evidences confirming that oxidative stress manifest as a consequence of the first insult, which turns out later to become the cause of epileptogenesis [34], other studies support the influence of oxidative stress in epilepsy. In accordance with Patel [34], oxidative stress is the cause or consequence of epileptic seizures.

Protein oxidation is an irreversible oxidative damage, considered to be a marker for severe oxidative stress [28]. Our results demonstrated increased levels of protein carbonyl when compared with the epilepsy vs. control group. Accordingly, Sudha et al. [35] described protein carbonyl increased levels in epilepsy patients than in controls. Of such importance, when analyzing MnSOD, the results suggested that the polymorphism plays an influence on its performance, in view that the homozygous VV epilepsy group demonstrated decreased activity when compared to AV and AA genotypes.

The apoptotic pathway occurs primarily through the extrinsic and intrinsic pathways [36]. In relation to intrinsic (or mitochondrial) pathway, the apoptosis can be initiated by cytokines such as IL-6. In this pathway, the mitochondria release cytochrome *c*, activating the caspase-3, leading to cell death [37]. Of note, our results demonstrated increased IL-6 and caspase-3 levels in the epilepsy group when compared to the control group. Accordingly, Peltola et al. [38] reported increased levels of IL-6 in plasma and cerebrospinal fluid (CSF) of epilepsy patients when compared to nonepilepsy patients. Increased caspase-3 in brain tissues has been found in animal models of epilepsy [12] and epilepsy patients [39]. Studies also relate increased serum caspase-3 with traumatic brain injury (TBI). However, caspase-3 has been scarcely explored in blood of epilepsy patients [40]. The Ala16Val MnSOD polymorphism also revealed a significant importance when associated with the genotype: the VV epilepsy group demonstrated increased levels of IL-6 and caspase-3 when compared to other genotypes (AV and AA). In this context, an interesting correlation between IL-6 vs. caspase-3 was observed. When compared to seizure type, a positive correlation was obtained when related to generalized seizures. Our results suggest that the Val allele has less efficiency, in view that the analyzed parameters were increased in the VV epilepsy group. Particularly, in the brain, these proteins can lead to the activation of caspase-3, inducing cell damage, such as DNA fragmentation [41]. In this study, we could also observe that in the epilepsy group, caspase-1 and IL-1*β* presented increased levels when compared to the control group. Accordingly, Shi et al. [42] published a study relating that the cytokine IL-1*β* has also been found to be significantly increased within the CSF in epilepsy pediatric population when compared to control group, suggesting the cytokine's important role in epilepsy initiation and progression. Of such interest, in view that the IL-1*β* has proconvulsant actions, it is likely that this cytokine is released by cells following an inciting event. In this sense, a complex activation, which includes the caspase-1 is a crucial step required for IL-1*β* release [43]. Accordingly, our results demonstrated an interesting correlation between IL-1*β* and caspase-1 in the epilepsy group–generalized seizures (VV genotype), corroborating with previous studies, emphasizing the great importance of Ala16Val MnSOD SNP seizure type in the obtained results. Still regarding to seizure type, it comes important in view that in generalized seizures, the disorders would be widespread throughout the brain [44]. When related to genetic polymorphism of MnSOD, we found some important results when analyzed AA, AV, and VV genotypes in the epilepsy group. It was observed a decreased MnSOD activity in the VV epilepsy group when compared to the AA epilepsy group. In this sense, our results are in accordance with that the ValVal could be less efficient than the AlaAla genotype to control the oxidative stress [17]. The V allele presents increased superoxide radical levels than the A allele due to its lower efficiency to dismutate this molecule into H_2_O_2_ [45]. Accordingly, the superoxide anion (O_2_^−^) is the central mediator of oxidative stress, this anion could lead to mitochondrial destabilization resulting in cell apoptosis activation [46]. Although the small sample size of the study, there are few studies indicating the association/influence of Ala16Val MnSOD polymorphism in epilepsy [25, 47]. We found some important associations with inflammatory, apoptotic, and oxidative stress biomarkers, suggesting that the Ala16Val MnSOD polymorphism has an important role on neuroinflammation maintenance and its consequences.

## 5. Conclusion

Finally, the result demonstrated influence of Ala16Val MnSOD polymorphism, mainly of VV genotype in epilepsy patients. Accordingly, studies have shown that the polymorphism of some genes may be related to the efficacy, tolerability, and action of antiepileptic drugs [48, 49].

In this sense, prolonged seizures or status epilepticus in epilepsy patients can become a serious problem due to their consequences on the quality life from this population [50]. Seizures can have devastating consequences and, as result, suffer bodily injury requiring hospitalization. Others have shortened life span due to the increased risk of unexpected sudden death that is associated with uncontrolled seizures [50–52]. Studies have shown that patients with epilepsy can be significant neuropsychological, psychiatric, and social impairments that limit employment, reduce marriage rates, and decrease quality of life [51–53]. Thus, genetic polymorphism becomes an “ally” to help discover the cause of drug refractoriness and provides insight into the type and magnitude of clinic-laboratorial manifestations may have across individuals, helping to determine the best treatment and improve the quality of life of patients with neurological diseases, such as epilepsy.

## Figures and Tables

**Table 1:** Characteristics of epilepsy and control groups.

Characteristics	Epilepsy (*n* = 47)	Control (*n* = 43)	*p* value
Gender			
Male	22 (46,8%)	23 (53,4%)	0.5
Female	25 (53,1%)	20 (46,5%)	
Mean age (years old)			
Male	36	39	0.6
Female	33	42	
Antiepileptic drugs (AEDs)			
Monotherapy	15 (31,9%)		
Polytherapy	32 (68,1%)		

**Table 2:** MnSOD Ala16Val genotype frequencies in epilepsy and control groups.

MnSOD SNP	Epilepsy	Control	*p* value
Genotypes			
AA	15 (31.9%)	17 (39.5%)	
AV	10 (21.2%)	14 (32.5%)	0.1
VV	22 (46.8%)	12 (27.9%)	

**Table 3:** Correlation among inflammatory, apoptotic, and DNA damage parameters with VV genotype in the epilepsy group and epilepsy group with generalized seizures.

Correlation	*r* value	*p* value
VV genotype–epilepsy group		
IL-1*β* vs. caspase-1	0.7	<0.001
IL-6 vs. caspase-3	0.5	<0.05
VV genotype–epilepsy group (generalized seizures)		
IL-1*β* vs. caspase-1	0.7	<0.05
IL-6 vs. caspase-3	0.6	<0.05

## Data Availability

The data availability data used to support the findings of this study are included within the article.
